# How the Commonwealth of the Northern Mariana Islands stalled COVID-19 for 22 months and managed its first significant community transmission

**DOI:** 10.5365/wpsar.2023.14.1.965

**Published:** 2023-01-25

**Authors:** Dwayne Davis, Stephanie Kern-Allely, Lily Muldoon, John M Tudela, Jesse Tudela, Renea Raho, Heather S Pangelinan, Halina Palacios, John Tabaguel, Alan Hinson, Guillermo Lifoifoi, Warren Villagomez, Joseph R Fauver, Haley L Cash, Esther Muña, Sean T Casey, Ali S Khan

**Affiliations:** a Commonwealth Healthcare Corporation, Saipan, Commonwealth of the Northern Mariana Islands, United States of America.; b Pacific Island Health Officers Association, Honolulu, HI, United States of America.; c University of California San Francisco, San Francisco, CA, United States of America.; d College of Public Health, University of Nebraska Medical Center, Omaha, NE, United States of America.; e World Health Organization Regional Office for the Western Pacific, Manila, Philippines.; f School of Population Health, University of New South Wales, Sydney, New South Wales, Australia.

## Abstract

**Objective:**

The Commonwealth of the Northern Mariana Islands (CNMI) is a remote Pacific island territory with a population of 47 329 that successfully prevented the significant introduction of coronavirus disease (COVID-19) until late 2021. This study documents how the response to the introduction of COVID-19 in CNMI in 2021 was conducted with limited resources without overwhelming local clinical capacity or compromising health service delivery for the population.

**Methods:**

Data from COVID-19 case investigations, contact tracing, the Commonwealth’s immunization registry and whole genome sequencing were collated and analysed as part of this study.

**Results:**

Between 26 March 2020 and 31 December 2021, 3281 cases and 14 deaths due to COVID-19 were reported in CNMI (case fatality rate, 0.4%). While notification rates were highest among younger age groups, hospitalization and mortality rates were disproportionately greater among those aged > 50 years and among the unvaccinated. The first widespread community transmission in CNMI was detected in October 2021, with genomic epidemiology and contact tracing data indicating a single introduction event involving the AY.25 lineage and subsequent rapid community spread. Vaccination coverage was high before widespread transmission occurred in October 2021 and increased further over the study period.

**Discussion:**

Robust preparedness and strong leadership generated resilience within the public health sector such that COVID-19 did not overwhelm CNMI’s health system as it did in other jurisdictions and countries around the world. At no point was hospital capacity exceeded, and all patients received adequate care without the need for health-care rationing.

In terms of vulnerabilities to infectious disease epidemics, the Pacific island countries and areas (PICs) have some unique advantages and disadvantages. Their remote location facilitates application of border control measures, and their low populations are often below the required threshold for the establishment of many epidemic-prone diseases. On the other hand, once an infectious disease is introduced, the populace is prone to explosive outbreaks and responses are often hampered by limited availability of health-care personnel and facilities, as well as supply chain constraints. Moreover, the islands – especially those with a high level of dependency on tourism income like the Commonwealth of the Northern Mariana Islands (CNMI) – can only remain closed off for so long without incurring a negative economic impact. Given that several PICs were able to effectively protect their population from the 1918–1919 influenza outbreak by introducing strict quarantine measures, (*1*) it is not surprising that this strategy was adopted by many at the start of the novel coronavirus disease (COVID-19) pandemic. Success among the PICs, however, has been variable. (*2*, *3*)

CNMI is a commonwealth in political union with the United States of America (USA) in the western Pacific Ocean, consisting of 14 tropical islands stretching over 400 nautical miles (740 km). More than 90% of the Commonwealth’s population lives on the island capital of Saipan (area, 46.5 square miles or 115 km^2^). Of the 13 other islands, only Rota and Tinian have a significant population. In Saipan, there is a single 86-bed hospital with four intensive care beds, five private clinics, and approximately 200 licensed physicians and advanced practice providers. CNMI has a shortage of health-care professionals, with the nurse-to-patient ratio in the hospital sometimes reaching levels of 1:7. (*4*) The semi-autonomous Commonwealth Health care Corporation (CHCC), an integrated health-care and public health system, serves as the Department of Health.

In response to reports of a novel coronavirus disease spreading in China, CNMI adopted a strict border policy in February 2020, which facilitated the identification and isolation of travel-associated cases. (*5*) The first community cases were identified on 26 March 2020 with limited further transmission. After eliminating local transmission in 2020, CNMI experienced its next community outbreak, again comprising only a small cluster of cases, in March 2021. A larger, more prolonged outbreak occurred at the end of 2021, extending into 2022. Before this large outbreak, CNMI’s leadership had time to obtain adequate resources, train personnel and deliver a community-based vaccination campaign. Thus, by the time of the first significant community spread, CNMI was uniquely protected; the case fatality rate was low and there was sufficient capacity within the health-care system to cope with increased case numbers as a result of the importation of both the Delta and Omicron variants of concern (VOCs).

The objective of this study is to describe CNMI’s adaptive public health response, which included strong border measures, contact tracing and a successful vaccination campaign, and its impact on COVID-19 transmission, morbidity and mortality. We also describe the characteristics and genomic epidemiology of COVID-19 cases in CNMI.

## Methods

### Data sources and case definitions

Laboratory-confirmed cases were reported to the COVID-19 Communicable Disease Investigation team who then conducted detailed case investigations, contact tracing and additional monitoring activities. Persons under investigation (PUI) were individuals with suspected COVID-19 based on clinical presentation. COVID-19 vaccination data were recorded by the Commonwealth’s immunization tracking system, WebIZ. (*6*) Age-group population estimates (denominators for vaccine coverage calculations) were extrapolated from the US Census Bureau’s International Database (IDB) 2020 age pyramid using the 2020 total census population of 47 329. (*7*, *8*) Racial and ethnic proportions of the population were sourced from the 2010 US Census. (*9*)

### Public health response

The CHCC led the public health response. In accordance with Public Law 13–63, the Territorial Health Official coordinated territorial leadership, with the support of the Governor and his COVID-19 Task Force, chaired by the Director of Hospital and Public Health Preparedness. (*10*)

### Community and hospital-based testing

Community-based testing evolved during the pandemic, especially after the start of the larger COVID-19 outbreak in October 2021. By late 2021, daily nucleic acid amplification tests (NAATs) were conducted by appointment, while antigen-based surveillance testing was performed on an as-needed basis at fire stations and quarterly in schools. Diagnostic testing was conducted for PUIs or symptomatic persons in quarantine at a community COVID-19 site using US Food and Drug Administration (FDA)-approved rapid antigen tests. All patients presenting to hospital with symptoms (or close contacts of positive cases) were tested using NAATs. Also, all health-care workers at CHCC were offered NAATs weekly.

### Contact tracing, isolation and quarantine

For the first 3 months of the October 2021 outbreak, all laboratory-confirmed cases were questioned about their recent contacts (during the 3 days before their symptom onset or positive test result). Isolation and quarantine periods followed contemporaneous US Centers for Disease Control and Prevention (CDC) guidelines. All cases and contacts were housed in government facilities – three contracted hotels – until this became logistically and financially untenable in November 2021 due to the high volume of cases. From then onwards, only symptomatic cases, those at higher risk of severe COVID-19 outcomes or those whose household was not completely vaccinated were required to complete isolation in government-managed facilities, while other cases completed isolation at home. All quarantined contacts were tested twice: once when identified as a contact and then again after completion of quarantine.

### Point-of-entry screening

Point-of-entry (POE) screening evolved during the pandemic as the science behind adequate quarantine and testing strategies progressed, additional testing became available and vaccination rates increased. Revisions to POE protocols were aligned with external recommendations and US CDC guidelines.

Beginning in March 2020, all arriving travellers were quarantined in a government-contracted quarantine hotel for 14 days and then tested before their release. From May 2020 onwards, all travellers were additionally tested on arrival. By July 2020, all visitors were required to complete an online travel registration form 72 hours before their entry into CNMI, quarantine at a government facility for 5 days (or at home for residents) and test negative for COVID-19 before release. For visitors who refused to be tested, the quarantine period was extended to 14 days. In August 2020, in a reaction to rising COVID-19 case numbers in the USA and nearby Guam, all travellers had to quarantine in a government facility for 5–7 days, depending on their vaccination status. By June 2021, rapid antigen testing was made available at POEs for arrival testing. After the identification of community cases in late October 2021, the quarantine period for all travellers was changed to 5–10 days, depending on vaccination status. Protocols were again adjusted in November 2021, allowing fully vaccinated travellers to complete quarantine under active surveillance outside of a government facility. However, travellers were required to take a NAAT on day 5 for clearance from self-quarantine. Throughout the acute period of the pandemic, qualified essential workers were granted modifications to entry requirements but only after submitting to a rigorous CHCC approval process.

### Health facility preparedness

Seventeen hospital rooms were upgraded to COVID-19 isolation rooms and fitted with air scrubbers and ultraviolet lights (one for labour and delivery, two for obstetrics, two for paediatrics and 12 for medical cases). In early 2021, a 25-bed Alternative Care Site (ACS) was established at a local hotel to expand bed capacity for less severely ill COVID-19 patients and as a step-down unit for those hospitalized in the main hospital. The ACS also supplied specialized services (e.g. haemodialysis) for patients in isolation. All COVID-19 patients were assessed for risk factors for severe disease and offered monoclonal antibody treatment at the ACS.

During the October 2021 outbreak, to overcome difficulties in sharing information with patients without access to phones and Wi-Fi, the COVID-19 Task Force established a physical community centre where patients could be tested, treated, and receive their test results and health advice.

### Community-based vaccination

From December 2020, all adults in CNMI were offered COVID-19 vaccines in line with the contemporaneous US CDC guidelines; as vaccines were authorized for use in children, vaccination was extended to those aged > 5 years. A community-based approach was used to maximize vaccine uptake, supplemented by a government mandate for all health-care workers and government employees. A directed “house-to-house” outreach campaign for vaccination and boosters targeting high-risk and low-turnout communities proved highly effective. Other initiatives included the “Road to 80” campaign, the aim of which was to fully vaccinate 80% of the population against COVID-19. The campaign ran from July to September 2021 and offered raffle prizes to any CNMI resident who had received the first dose of any available vaccine. Vaccine supply, technical assistance and logistics were provided by the US CDC.

### Diagnostics

Samples for laboratory diagnostics comprised either nasopharyngeal swabs placed in universal transport media for NAATs (except for ID NOW [Abbott Laboratories, Abbott Park, IL, USA] which uses a disposable dry swab) or nasal swabs for antigen detection assays. Initially, testing was performed at Guam Public Health Laboratory. From mid-April 2020 onwards, once FDA authorization had been obtained, NAAT was conducted locally in CNMI using the DiaPlexQ novel coronavirus detection kit (SolGent Co., Ltd, Daejeon, Republic of Korea). Travellers, PUIs presenting to the hospital and individuals testing positive with the DiaPlexQ assay were tested by NAAT with either ID NOW or GeneXpert; all positives were considered laboratory-confirmed cases. PUIs and symptomatic persons from the community in quarantine were tested using BinaxNOW (Abbott Laboratories) rapid antigen test and considered laboratory-confirmed cases if their test was positive. The COVICHEK antigen kit (WiZChem Co., Ltd, Kangwon, Republic of Korea) was used for community-based surveillance, but all positive test results were confirmed by NAAT or BinaxNOW assay.

### Genomic and phylogenetic analysis

Specimens positive for severe acute respiratory syndrome coronavirus 2 (SARS-CoV-2) were sent to the US CDC Division of Viral Diseases for whole genome sequencing. Samples were sequenced using Illumina (San Diego, CA, USA) platforms and consensus sequence genomes were uploaded to GISAID. Nextstrain (*11*) was used to conduct all phylogenetic analyses. Between December 2019 and July 2022, 13 090 phylogenetic analyses were conducted: 2945 genomes from CNMI and 10 145 contextual genomes, with preference given to specimens from countries with geographical proximity to CNMI, including Guam, Indonesia, Japan, Malaysia, Papua New Guinea, the Philippines, the Republic of Korea and the USA. Following the standard Nextstrain Augur (*12*) pipeline, nucleotide alignment was conducted with MAFFT, (*13*) maximum-likelihood phylogenetic trees were created with IQ-TREE2, (*14*) time-resolved phylogenetic trees were created with TreeTime (*15*) and results were visualized using Auspice. Nodes on the phylogenetic tree were annotated to indicate how the cases were identified (i.e. through travel screening, hospitalization, community testing or contact tracing) and inferred dates estimated. The inferred date is the date when a specific SARS-CoV-2 genotype arose, which may not necessarily be the date it was introduced. This date, by definition, must be earlier than when the first case attributable to a given genotype was detected.

### Statistical analysis

Frequencies for categorical variables were tabulated. Crude event rates were calculated by dividing the number of infections, hospitalizations or deaths by the total population (or vaccination status subgroup). Risk ratios were calculated for the risk of hospitalization by vaccination status and were adjusted for age and sex. All analyses were performed using R version 4.1.1.

## Results

### Descriptive epidemiology

**Fig. 1:**
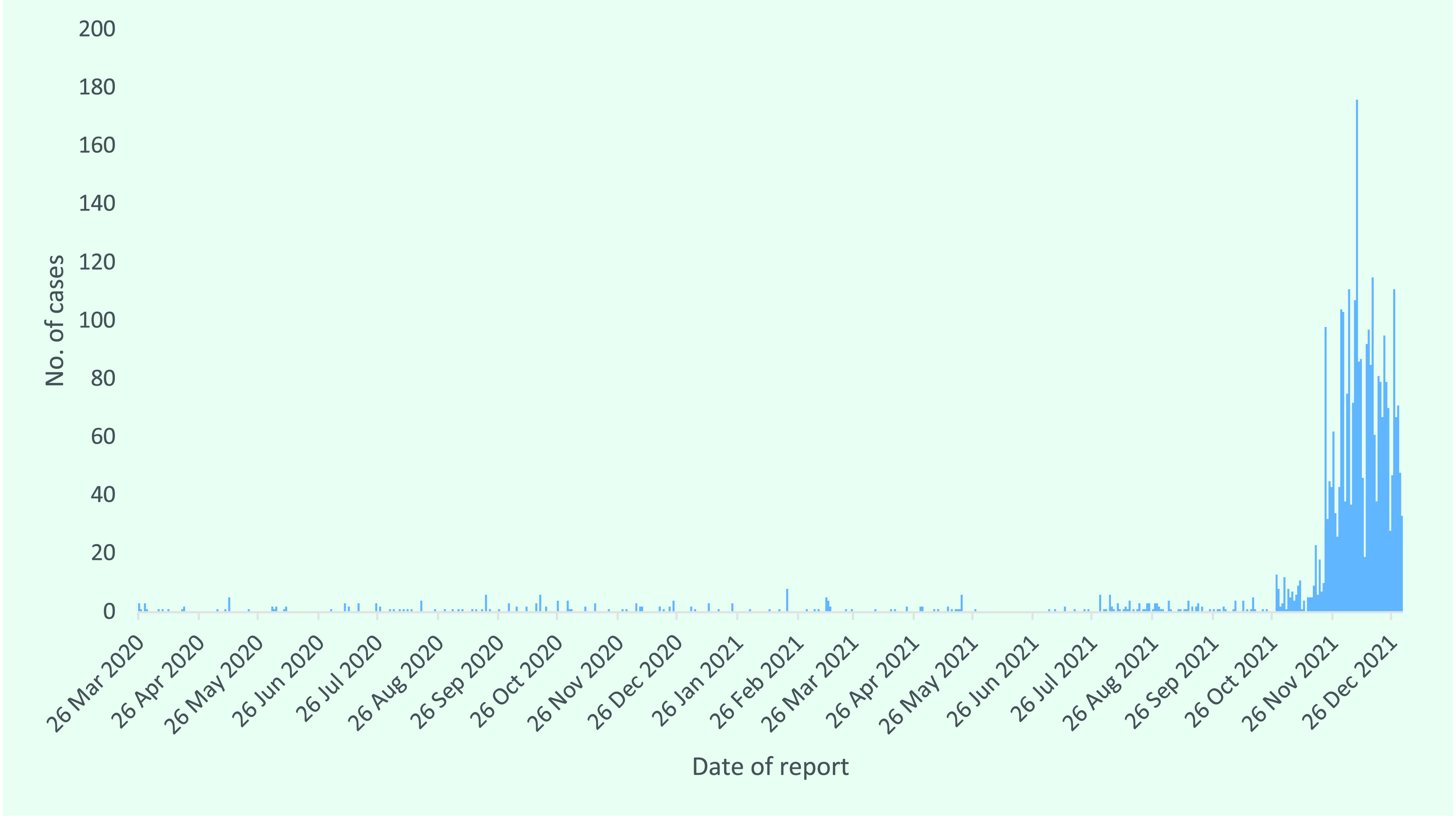
Daily number of laboratory-confirmed COVID-19 cases, Commonwealth of the Northern Mariana Islands, 26 March 2020–31 December 2021 (N = 3281)

From 26 March 2020 to 31 December 2021, 3281 cases of COVID-19 were recorded in CNMI (**Fig. 1**). There were 87 hospitalized cases (2.6%) and 14 deaths listing COVID-19 as either a cause of death or a contributing condition (case fatality rate, 0.4%; 30 deaths per 100 000 population). Nearly one third of cases (30.8%; *n* = 1009) reported symptoms characteristic of COVID-19 (e.g. fever, cough, shortness of breath, anosmia, ageusia). By December 2021, approximately 7% of CNMI’s population had been infected with COVID-19. Virtually all cases were identified on Saipan; just nine cases (0.3%) were identified among residents of Tinian.

Between March 2020 and October 2021, the period between the first case notification and the start of the larger community outbreak, CNMI recorded just 291 cases (**Fig. 1**): 250 were identified from travel quarantine, 26 from contact tracing (primarily in recent travellers), 8 from hospital testing and 7 from community-based testing.

[**insert** Figure 1]

After the introduction of COVID-19 in March 2020 and subsequent elimination of community transmission by April 2020, the next community outbreak of COVID-19 comprised 11 cases, the first of which was identified on 12 March 2021 through outbound travel testing and the last on 17 March 2021. The much larger community outbreak started on 28 October 2021, with the first cases identified through school-based testing. At the time of writing (early 2022), this outbreak was still ongoing, albeit at lower levels.

**Fig. 2:**
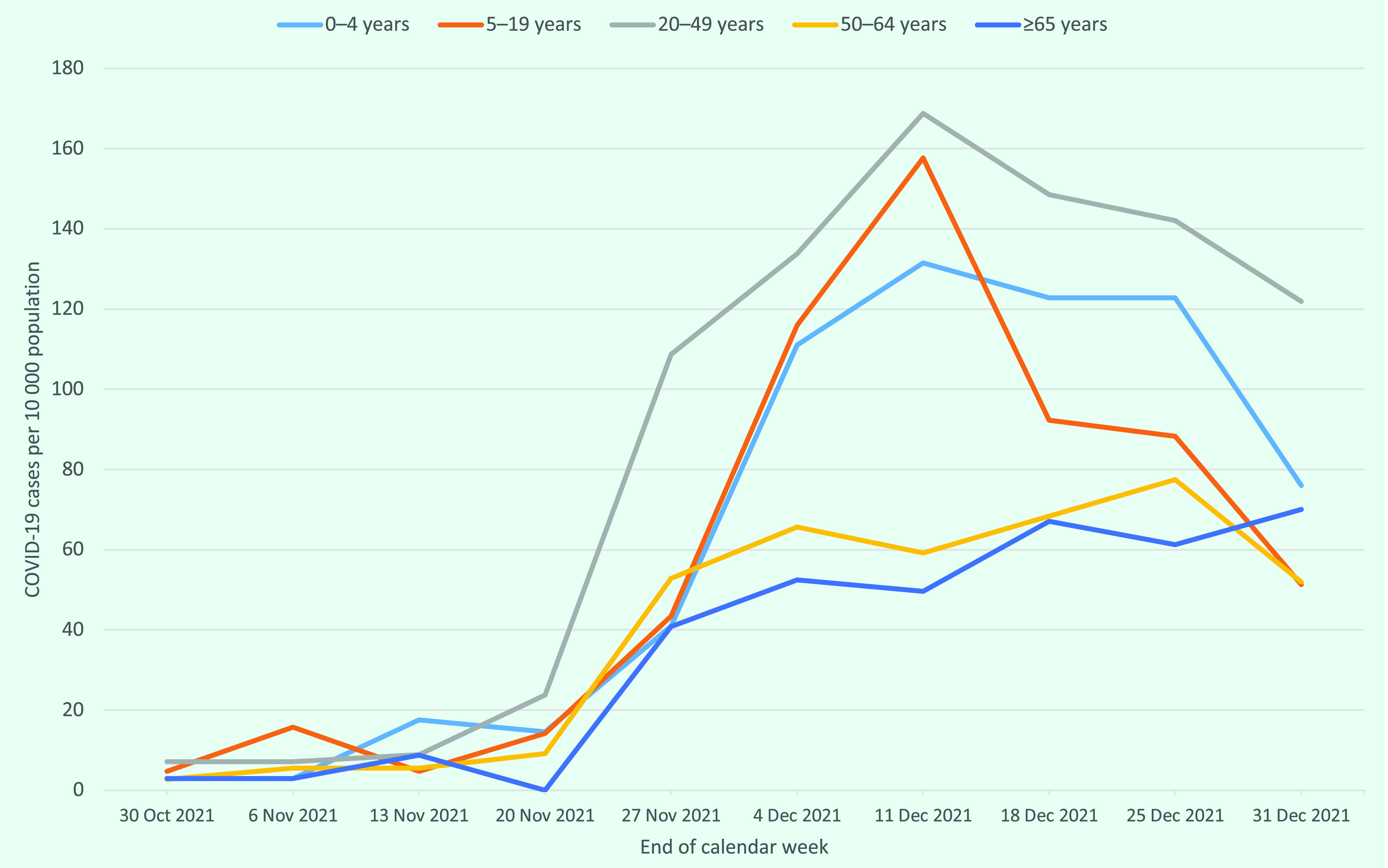
Weekly notification rates of COVID-19 by age group, Commonwealth of the Northern Mariana Islands, 29 October–31 December 2021

The mean age of all COVID-19 cases was 31 years (range: 0–95 years) and 53.9% were in men. Notification rates during the study period were highest in those aged 20–49 years (963.4 cases per 10 000 persons; *n* = 1621), followed by those aged 0–4 years (680.9 cases per 10 000 persons; *n* = 233) and those aged 5–19 years (622.8 cases per 10 000 persons; *n* = 790). The lowest rates were seen in the oldest age group, those aged ≥ 65 years (399.8 cases per 10 000 persons; *n* = 137), and the next oldest group, those aged 50–64 years (455.7 cases per 10 000 persons; *n* = 400). Weekly notification rates increased especially rapidly in people aged < 50 years from the week of 20 November to the week of 11 December 2021; rates in those aged > 50 years increased more gradually over the same time frame (**Fig. 2**).

[**insert** Figure 2]

The mean age of hospitalized cases was 49 years (range: 0–95 years). Hospitalization rates were highest among those aged ≥ 65 years (61.3 hospitalizations per 10 000 persons; *n* = 21), followed by those aged 50–64 years (24.6 per 10 000 persons; *n* = 27) and those aged 20–49 years (19.6 per 10 000 persons; *n* = 33). Hospitalization rates were low in children, with 8.8 per 10 000 persons (*n* = 8) among those < 5 years and 2.4 per 10 000 persons (*n* = 3) in those aged 5–19 years. Over two fifths of hospitalized cases (43.7%) were admitted through the emergency department  (*n* = 38). After a medical assessment, 378 (11.5%) patients received monoclonal antibodies.

**Fig. 3:**
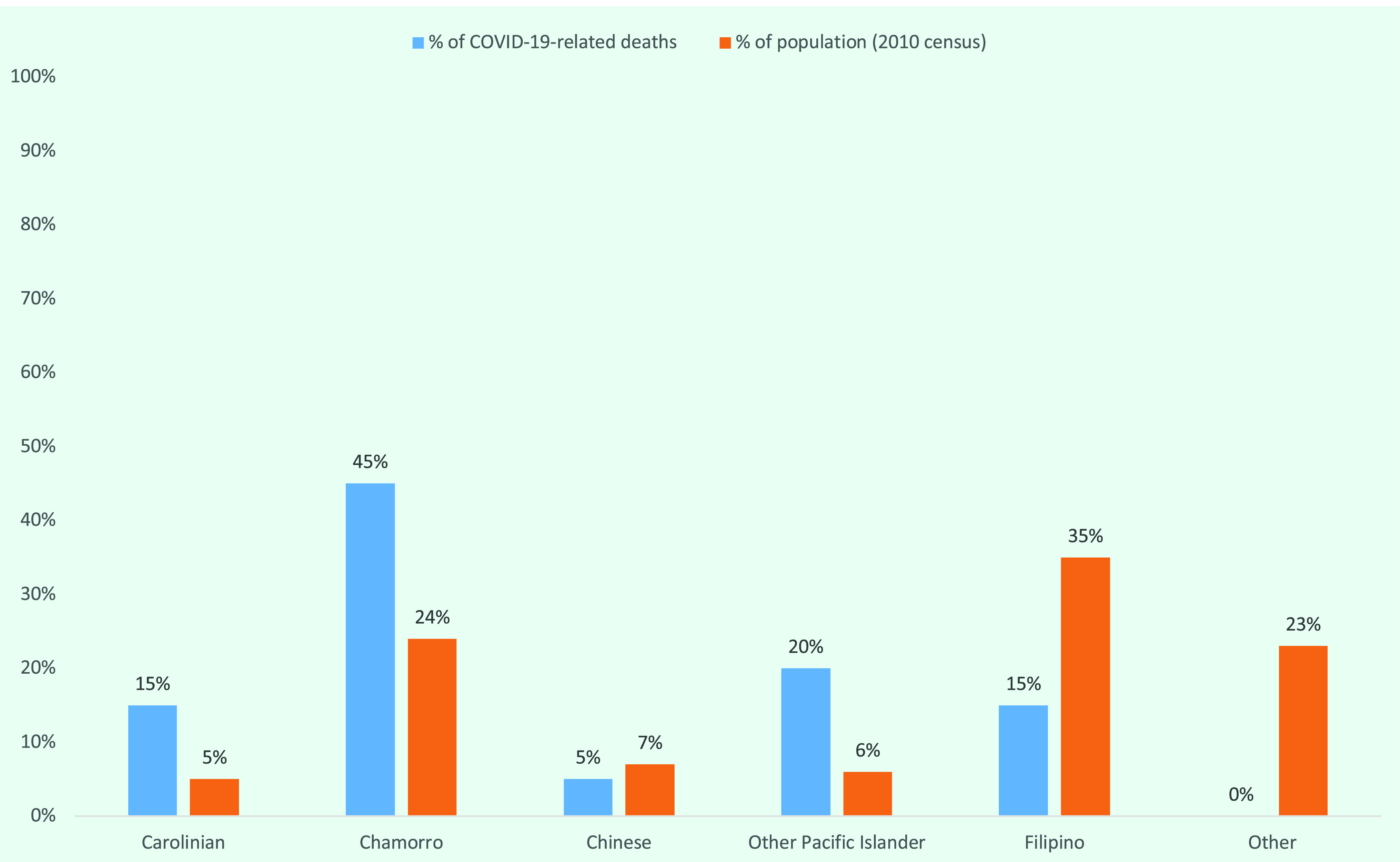
COVID-19-related deaths by race/ethnicity compared to 2010 Census population, Commonwealth of the Northern Mariana Islands, March 2020–December 2021 (N = 14)

All but one of the 14 COVID-19-related deaths occurred in people aged > 50 years; there was one death in a 44-year-old. Relative to the 2010 US Census population estimates, Carolinian, Chamorro and other Pacific Islanders were overrepresented in the deaths (**Fig. 3**). Of the 14 deaths, nine (64.3%) were in unvaccinated individuals.

[**insert** Figure 3]

Two thirds of cases had received at least one dose of a COVID-19 vaccine; 60.2% (*n* = 1975) were fully vaccinated and 5.7% (*n* = 188) were partially vaccinated. Of the remainder, 25.7% (*n* = 845) were unvaccinated and 8.3% (*n* = 273) were ineligible for COVID-19 vaccination. Cases who were unvaccinated had a risk of hospitalization 2.64 times (95% confidence interval [CI]: 1.71–4.07) higher and a risk of death 3.63 times (95% CI: 1.14–11.55) higher than those who were fully vaccinated.

Almost half of all reported cases (46.4%; *n* = 1524) were identified through contact tracing. Nearly another third were identified from community-based testing (27.8%; *n* = 912), with the remainder of cases coming from hospital testing (16.3%; *n* = 535) and incoming travellers (9.6%; *n* = 314). A total of 14 672 contacts were actively monitored by public health staff, with an average of 4.5 contacts monitored per case (range: 0–48).

### Genomic epidemiology

Of the 3281 COVID-19 cases, genomes were sequenced from 2945 (89.8%). Ten of the 11 cases from the March 2021 community outbreak were sequenced, revealing that this cluster not only comprised viruses in the B.1.2 Pango lineage (*16*) but was also a monophyletic cluster of largely identical genomes. Genomes from this cluster were direct descendants of two cases identified through travel screening on 2 February 2021.

**Fig. 4:**
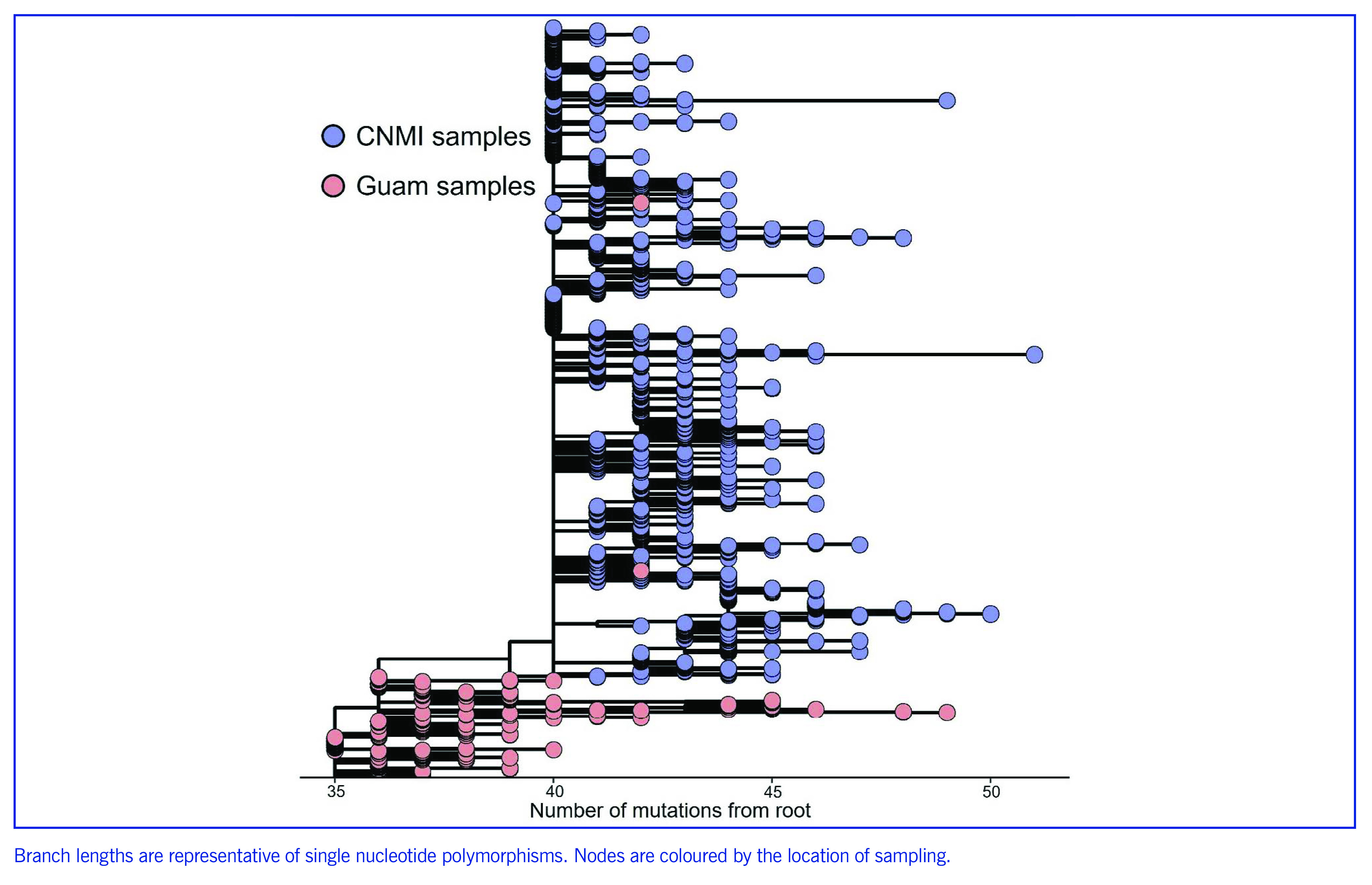
Phylogenetic tree of the large COVID-19 outbreak of Delta lineage AY.25, Commonwealth of the Northern Mariana Islands, July 2021–January 2022

Genome sequencing of cases from the larger October 2021 community outbreak also revealed a large monophyletic cluster of a virus from the AY.25 (Delta variant) Pango lineage (**Fig. 4**), suggestive of a single introduction event. Many of the samples from the early cases fell into a large polytomy of identical genomes with subsequent branches coming from the polytomy, consistent with rapid spread following the introduction. Three mutations separate the internal CNMI cluster, and this branch has an estimated inferred date of 27 July 2021 (CI: 4 June–5 August 2021).

Phylogenetic analysis further showed that the monophyletic cluster of CNMI genomes were direct descendants of genomes sequenced from Guam earlier in 2021 (**Fig. 4**). Genomes collected from Guam in late July–early August 2021 were the most recent common ancestor of the CNMI cluster. This suggests that the large outbreak in CNMI was caused by an introduction of a single genotype in the AY.25 lineage, most likely from Guam. Supporting evidence comes from contact tracing data, which dated the earliest symptom onset in a community case to early October 2021. This individual reported recent contact with a traveller with “essential worker” status from Guam before their symptom onset.

[**insert** Figure 4]

### Vaccination coverage

**Fig. 5:**
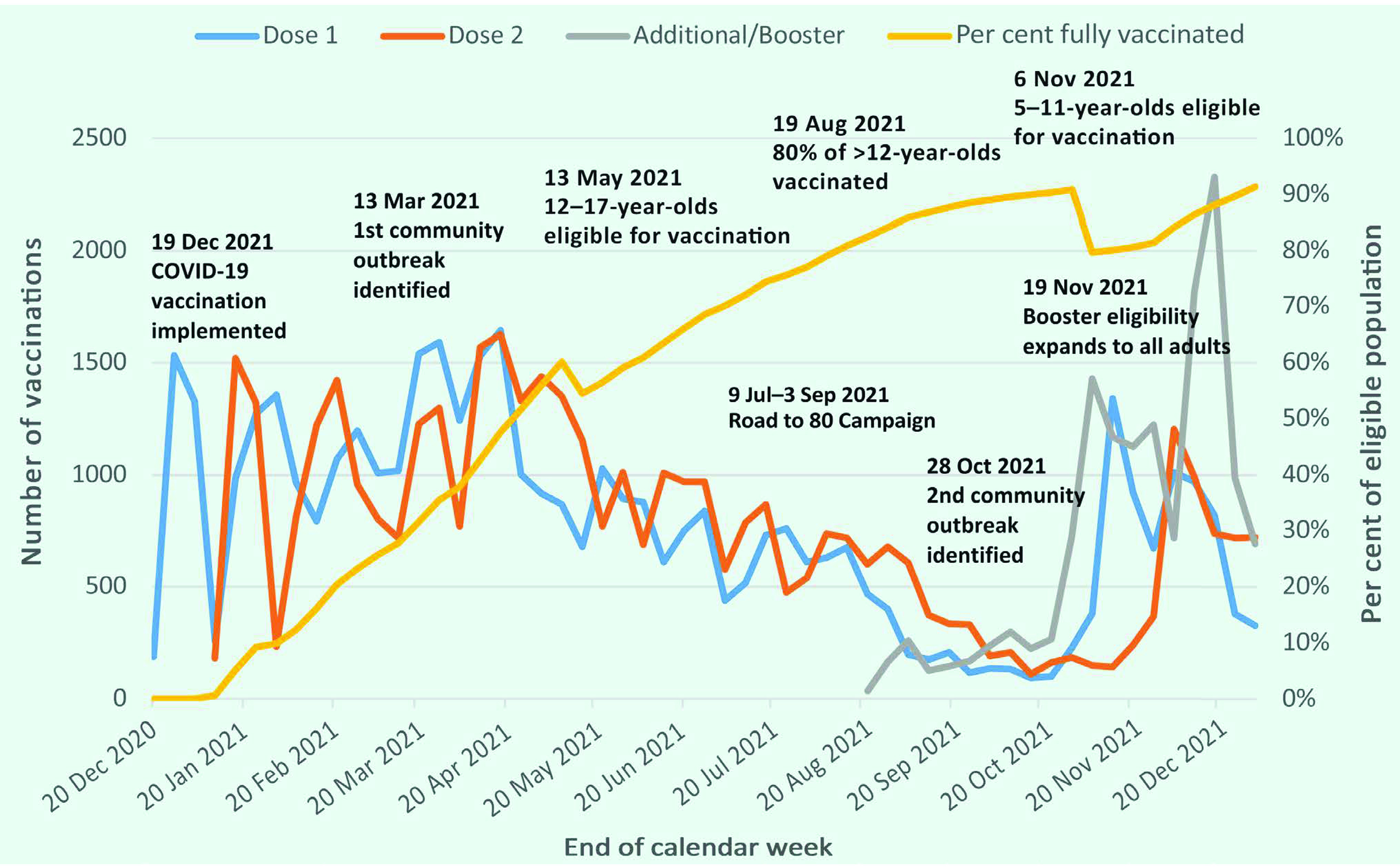
Timeline of the COVID-19 vaccination programme, Commonwealth of the Northern Mariana Islands, 19 December 2020–2 January 2022

By 31 December 2021, 96 745 vaccine doses had been administered in CNMI – 82 145 doses of Pfizer-BioNTech, 13 348 doses of Moderna, 1245 doses of Johnson & Johnson/Janssen and seven unknowns. Before the October 2021 outbreak, vaccine coverage was 73.4% (*n* = 34 745) in the overall population and 90.4% of the vaccine-eligible population (i.e. those aged > 12 years). By 31 December 2021, vaccine coverage reached 84.8% in the overall population (*n* = 40 121) and 91.4% among the vaccine-eligible population (i.e. those aged > 5 years); 32.2% of those eligible (*n* = 14 140) were up to date with boosters (**Fig. 5**). Of note, the vaccine coverage rate among adults aged ≥ 65 years was > 99%.

[**insert** Figure 5]

## Discussion

CNMI demonstrated a well coordinated public health response to the COVID-19 pandemic. Initial efforts stalled major community transmission of COVID-19 for 22 months and provided a window of opportunity to prepare for the eventual community introduction of COVID-19 by implementing a vaccination campaign as well as measures to ensure preparedness and efficient use of federal and partner emergency health system resources.

In common with other PICs, CNMI’s initial response to COVID-19 relied on strict border controls; however, these were inadequate to identify and isolate all cases with POE screening. Nevertheless, the rapid containment of the small community outbreak of COVID-19 in March 2021, which was limited to just 11 cases, showed the ability of CNMI to successfully implement an elimination model of disease transmission for breakthrough cases.

As the pandemic progressed, and given the reality of increased border crossings, lapses in quarantine processes or testing, and shorter quarantine times as PICs titrated efforts to maintain tourism and protect their economies, most PICs had to face the inevitability of community transmission of COVID-19. Indeed, in CNMI, the large community outbreak in October 2021 was traced to close contact with an essential worker and the bypassing of the rigid quarantine system that had helped to prevent widespread disease introduction for almost 22 months.

The epidemiology of COVID-19 in CNMI mirrors that observed in other jurisdictions around the world, with case rates highest among younger age groups and rates of severe disease, hospitalizations and deaths highest in those aged > 50 years. (*17*) Given the high transmissibility of the Delta VOC (*18*) and the CHCC’s limited capacity to monitor a large number of cases, CNMI’s leadership when formulating its response to the October 2021 outbreak made the decision to scale back its resource-intensive contact tracing, quarantine and isolation measures. By this time, the vaccination programme had fully vaccinated 73.5% of the eligible population in CNMI, helping to keep hospitalization and mortality rates low and preventing the health-care system from being overwhelmed. Lessons learned from the success of the Delta response have since been used to inform the response to the Omicron variant which – unlike the situation in other countries where there was a gap between the Delta and Omicron waves – was also circulating in the community by December 2021.

In CNMI, unique challenges such as limited health-care facility capacity and delayed access to surge personnel and supplies served as drivers for aggressive preparedness and response actions. Strict POE protocols prevented widespread community transmission initially and delayed the worst of the impacts of COVID-19 while local efforts focused on learning, assembling and vaccinating. However, as POE restrictions were relaxed and more transmissible variants (Delta and later Omicron) overwhelmed contact tracing efforts, widespread community transmission ultimately occurred, and resources were redirected to other critical response efforts. Nonetheless, the alignment of political and health leadership with a community-based approach tempered many of the challenges faced on the US mainland, including overstretched hospitals and high mortality rates. The high vaccination rate, achieved in a multiethnic, multiracial community despite the spread of misinformation, was also a major contributor to the success of the response. CNMI has had relatively mild morbidity and mortality from this pandemic thanks to its model health response.
